# Analyzing the microstructure and related properties of 2D materials by transmission electron microscopy

**DOI:** 10.1186/s42649-019-0013-5

**Published:** 2019-11-04

**Authors:** Yun-Yeong Chang, Heung Nam Han, Miyoung Kim

**Affiliations:** Department of Materials Science and Engineering, Seoul National University, Seoul, 151-744 Korea

**Keywords:** Transmission electron microscopy, 2D materials, Transition metal dichalcogenide, Graphene, Van der Waals heterostructure

## Abstract

Two-dimensional materials such as transition metal dichalcogenide and graphene are of great interest due to their intriguing electronic and optical properties such as metal-insulator transition based on structural variation. Accordingly, detailed analyses of structural tunability with transmission electron microscopy have become increasingly important for understanding atomic configurations. This review presents a few analyses that can be applied to two-dimensional materials using transmission electron microscopy.

## Introduction

Two-dimensional materials are considered as new candidates for future electronic and optical low-dimensional materials. In particular, the most spotlighted materials are graphene and transition metal dichalcogenides (TMD). TMDs are materials of type MX_2_, where M represents transition metal atoms such as Mo and W and X represents chalcogen atoms such as S, Se, and others. In contrast to graphene, TMDs have band gaps (Manzeli et al. 2017). The properties of graphene and TMDs’ can be tuned using varying growth conditions. Especially for TMDs, the band gap can be modified by decreasing the thickness from a few layers to a monolayer, by creating defects or implantations on the pristine surface and inducing electron-beam irradiation on the surface. For example, the most investigated material, MoS_2_, shows an indirect band gap in its bulk state, but when the layers are decreased into monolayers, the band gap changes into a direct band gap (He et al. 2014). This leads to better photoluminescence efficiency (He et al. 2014; Wang et al. 2015).

Both graphene and TMDs form van der Waals bonds, which is a weak force of attraction between the layers when they are stacked into few layers. In bilayer 2D materials, the stacking sequence modifies the crystal symmetry and equilibrium distance, which affects the physical properties of 2D materials, such as band gap, phonon vibration frequency, and superconductivity (He et al. 2014; Yan et al. 2015). Recent studies on the manipulation of the interlayer sequence twist angle showed electron tunneling in the heterostructure of 2D materials (Ribeiro-Palau et al. 2018). For example, graphene showed a strong coupling effect when the bilayers were twisted at the angle of 1.1°, which is called the magic angle (Cao et al. 2018). Likewise, the TMD heterostructure demonstrates angle sensitivity in the formation of interlayer excitons (Rivera et al. 2015; Ribeiro-Palau et al. 2018).

Other 2D materials such as 1 T-TaSe_2_ and TaS_2_ have a unique characteristic imparted by Fermi nesting, the charge density wave (CDW) (Johannes and Mazin 2008). The CDW is the periodic modulation of the charge density related to the periodic lattice distortion, which is observed as periodic structural changes in the atomic sites (Hossain et al. 2017). This lattice distortion occurs in two different forms, i.e., the commensurate and incommensurate structures; commensurate structures have electron density in the rational multiple of lattice distortion, whereas incommensurate structures have periodicities in the irrational number (Chen et al. 2016). At low temperatures, the property of CDW presents the superconductivity when it orders. Because of such distinct characteristics of CDW, much effort has been made to elucidate the underlying mechanism (Castro Neto 2001; Calandra 2015).

To understand the characteristics described, information on the atomic configuration of 2D materials is essential. Transmission electron microscopy (TEM) is the most commonly used technique for obtaining structural information. In particular, the Z contrast nature of high-angle annular dark-field (HAADF) scanning transmission electron microscopy (STEM) imaging enables to discern each atom in the 2D layer. The well-known disadvantages of TEM, such as knock-on damage and resolution problems, have been overcome with the recent progress in aberration correction. Aberration-corrected TEM enables to image atomic configurations of 2D layered structures at low acceleration voltages, which significantly reduces knock-on damage. Moreover, the domain patterns that form between the van der Waals layers, such as moiré patterns, can be identified with TEM, and the CDW changes can also be depicted in the diffraction patterns.

This review presents the TEM observation of defects and implantation and structural changes of 2D layers. In addition, research regarding the microstructure of 2D materials with atomic resolution imaging and electrical property measurements is described.

### Observation of defects, interstitial sites, and single atoms in 2D materials

TEM has long been used to observe individual atoms, defects, and interstitial sites. Atomic-resolution information on the crystal structure of 2D materials by TEM has gained immense importance because the band structure of the material significantly changes in response to the defects, number of layers, and atomic configurations (Wang et al. 2012; Wang et al. 2017; Elibol et al. 2018). For example, MoS_2_ ferromagnetism is enhanced with the fabrication of atomic vacancies with electrons or ion beam. In addition, semiconducting MoTe_2_ showed a new mid-gap state in the band gap induced by imbedded quantum dots and quantum walls (Lin et al. 2016; Elibol et al. 2018). The defects also affect the localization of spin-orbit coupling in graphene. The coupling effect modifies the spin transport characteristics of TMD and graphene layers (Garcia et al. 2018). Furthermore, in specific graphene edge structures, a new band gap and spin state are induced when an electric field is applied (Wang et al. 2017). Because of the tunability of the electrical property with structure, the fabrication of materials with defects, interstitial sites, vacancies, and edge construction has become important.

The accurate identification of atomic configurations of 2D materials is the main issue in material tuning. Thus, effort has been made to clearly analyze the point or line defects in structures by using TEM. However, radiation-induced damage remains one of the main limitations of high-resolution transmission electron microscopy (HRTEM) of 2D materials (Meyer et al. 2012). To solve this problem, Garcia et al. (2018) investigated the e-beam irradiation effects on the MoS_2_ layer at different kV levels. They calculated the energy transfer from the electron beam to the material and compared it with the displacement threshold energy of Mo and S. At 200 kV and 120 kV, structural damage was observed, whereas the sample was stable at 80 kV. This result indicates that an electron beam of 80 kV is optimum for reducing structural damage to MoS_2_. This acted as the basis for the TEM analysis of the possible stable state. The difficulty of immaculate sample preparation is another limitation of TEM imaging. Rooney et al. (2017) compared microstructures using various sample preparation methods to observe the relationship between sampling methods and defect creation between layers. They applied density functional theory (DFT) calculations to determine the interlayer distance in the presence/absence of defects and compared them with the intensity profiles of real TEM cross-sectional views. The layers were more distant than the calculated results of the pristine surface, proving that defects or layer distortions can occur during sample preparation. These results indicated that sample fabrication inside a glovebox was the least damaging method of sample fabrication.

**Fig. 1:**
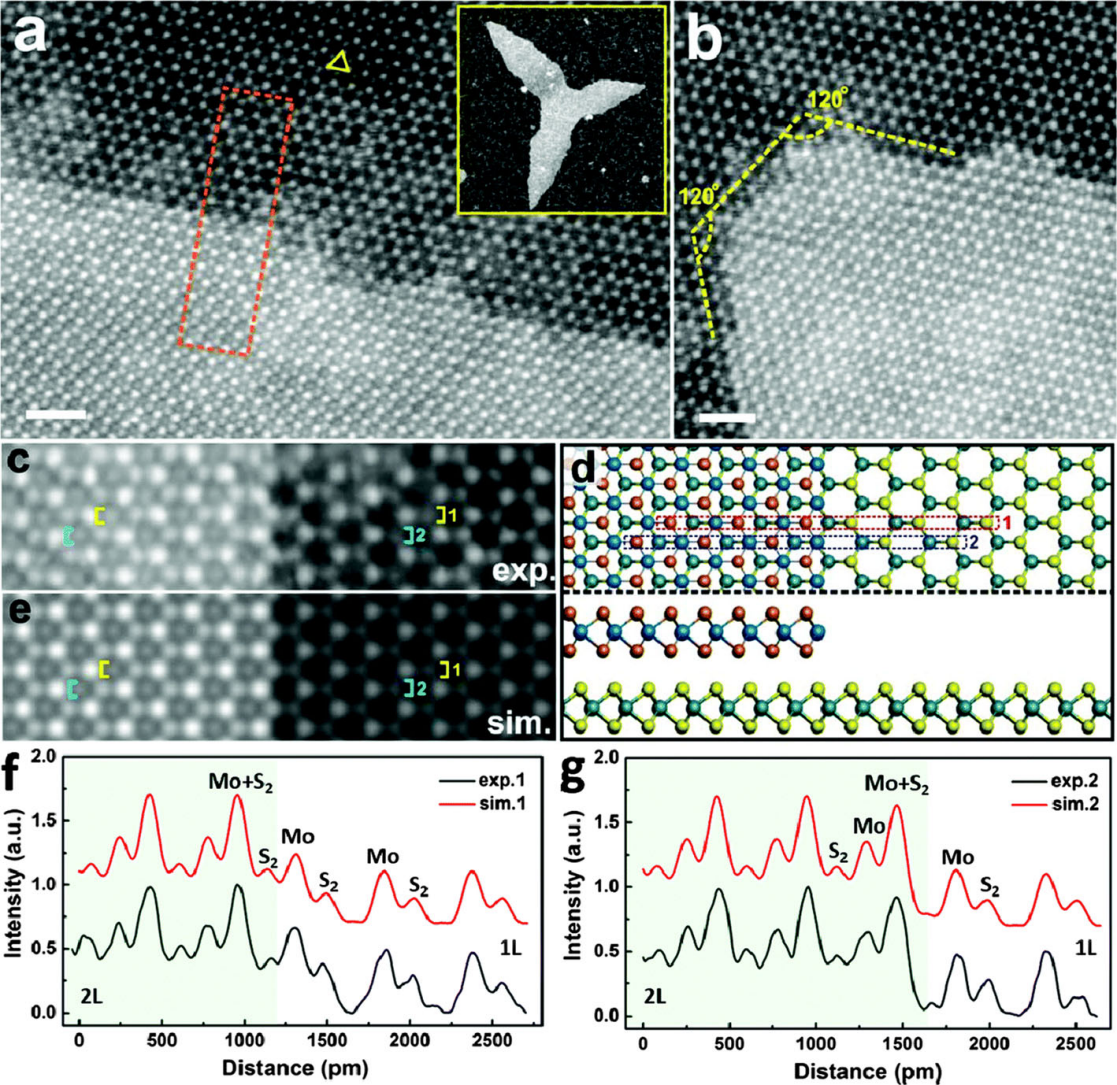
ADF-STEM images showing stacked bilayer MoS_2_. **a** ADF-STEM image of the tip region of stacked bilayer MoS_2_. **b** ADF-STEM image showing the extended bilayer MoS_2_ step edge. **c** Magnified image of the small region in (**a**). **d** Atomic model based on (**c**) showing both projection and side views. **e** Multi-slice ADF-STEM image simulation corresponding to the atomic model in (**d**). **f** Intensity line comparison between the simulated and experimental image of the site terminated with S_2_ atoms. **g** Intensity line comparison between the simulated and experimental image of the site terminated with Mo + S_2_ atoms. Reprinted from Wang et al. (2017) (*Nanoscale* 9, 13,060–13,068) with *Nanoscale*’s permission

To identify the accurate sites of atoms, vacancies, or other defects, simulation and intensity profile comparison has been combined with STEM HAADF imaging for better accuracy (Krivanek et al. 2010; Pham and Yeom 2016; Bangert et al. 2017). For example, in Fig. 1, an STEM image analysis comparing a simulated image and an experimental image provides information to discern the atoms at the terminated site (Wang et al. 2017). Similarly, the type of interstitial atoms are determined using a Z contrast comparison with a simulated image (Bangert et al. 2017). The HAADF image that best matches the atomic configuration relaxed by the DFT revealed the structural distortion in MoTe_2_ from the electron irradiation effect inside the TEM (Elibol et al. 2018). Atomic bonding near the Te vacancy was also studied by investigating the shift of atomic position in the image. Zheng et al. (2019) investigated the most predominant defect formation sites in WSe_2_ by examining HAADF images combined with DFT calculations of the vacancy formation energies. In their research, in contrast to previous expectations, Se vacancies were preferred to W vacancies. Moreover, Se sites were substituted by O in an amorphous structure. These defect formations and interstitial observations using TEM images explicated the structural state of 2D materials, which is important for their functional use.

### Observation of structural distortion

The thin layer property of 2D materials enables to induce their structural deformation in the domain scale, and this is commonly observed using TEM.

#### -2D layer phase structural transformation

**Fig. 2:**
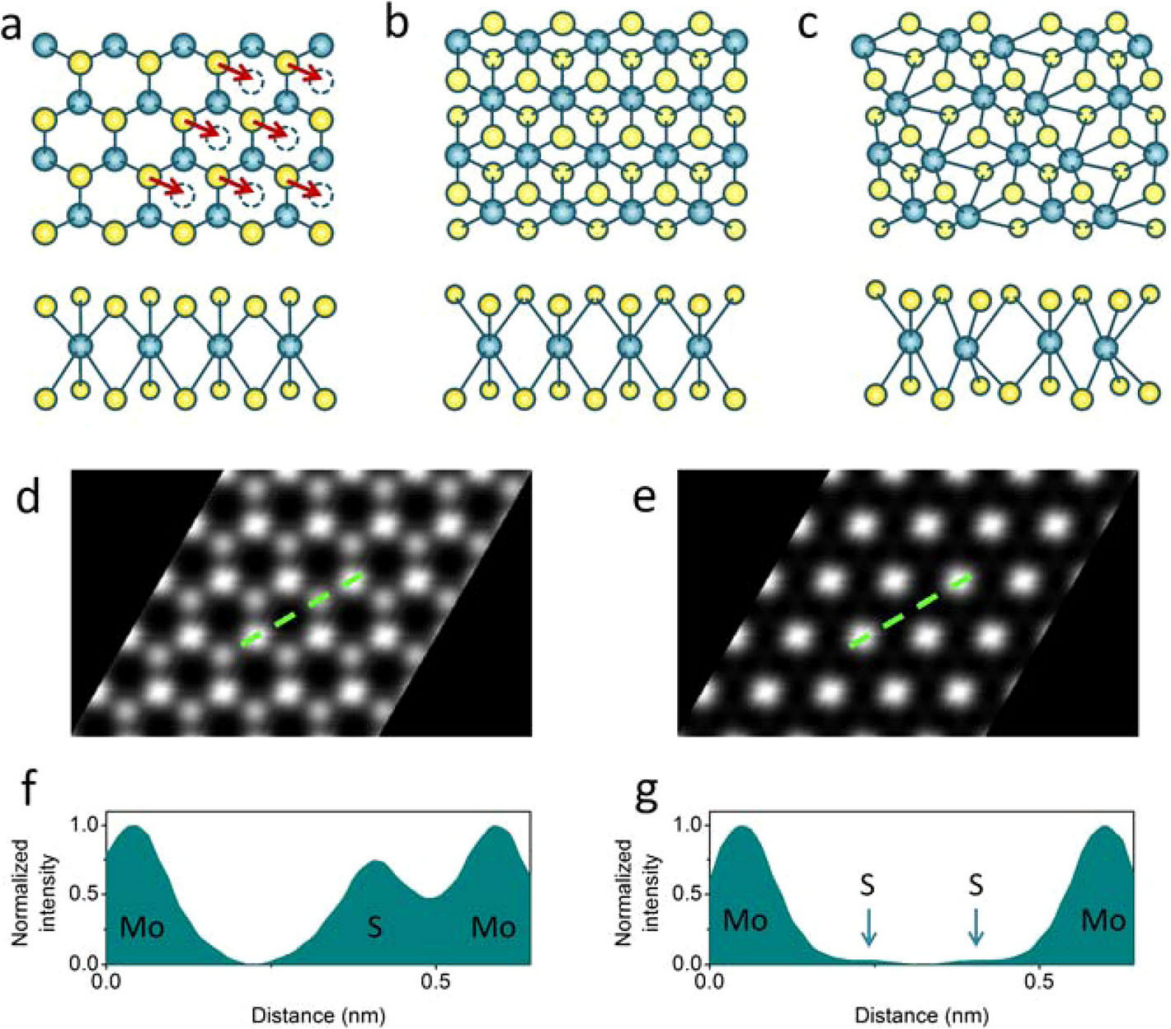
(**a**) 2H (the arrows show the local S glide inducing local transformation of the 1 T structure), (**b**) 1 T, and (**c**) 1 T’ phases viewed from the out-of-plane and in-plane axes. (**d** and **e**) Simulated HAADF STEM images of ideal (**d**) 2H and (**e**) 1 T phases. Reprinted from Eda et al. (2012) (*ACS Nano* 6, 7311–7317) with *ACS Nano*’s permission. (**f**, **g**) Intensity profile along the lines indicated in images **d** and **e** are shown in images **f** and **g**, respectively. bulk

Compared with graphene, TMD materials have few structural phases, mostly trigonal prismatic (2H) and octahedral (1 T). 2H is in the coordination of chalcogen atom in the same place in perpendicularly situating in the different layer (Fig. 2a), with the metal between them forming the ABA-like structure, and the 1 T structure is in the ABC stacking in which the chalcogen atoms are not in the same perpendicular line (Fig. 2b) (Manzeli et al. 2017; Gao et al. 2015). The electrical characteristics of each structure differ. 2H Mo- and W-based materials are semiconductors, and their photon absorption gap is 1–2 eV. The 2H structure, distorted 1 T phase, and 1 T’ (Fig. 2c) demonstrate semi-metallic characteristics (Huang et al. 2016; Li et al. 2016). These structures are discernable using the HAADF image (Fig. 2d and e) (Eda et al. 2012). The significance of structure-dependent electrical properties is increasing because of the changeability of the conductance in materials with the same stoichiometry. Transformations between the phases are induced by stimulating the material with electron beam irradiation. Elibol et al. (2018) reported that Te vacancies converted 1H structures into 1 T’ changes using STEM HAADF imaging and the radiation effect. The phase transformation leads to a more stable state, which minimizes the strain generated by the vacancies. The electron beam in TEM also activated inversion-domain formation and structural distortions in MoS_2_ (Ryu et al. 2016; Elibol et al. 2018). The S vacancies induced by electron beam irradiation during atomic resolution TEM accumulated and resulted in line defects. These line defects clustered and formatted the hold in the MoS_2_ (Ryu et al. 2016). In MoSe_2_, Se vacancies induced by radiation agglomerated into line defects and sequential atomic movement, filling the vacancies and resulting in triangular inversion symmetry. The formation mechanisms of phase changes in 2D materials have been more thoroughly understood with these studies.

#### -periodic lattice distortion-induced modifications

Periodic lattice distortion (PLD) has been observed in selected area electron diffraction (SAED) (Ishiguro et al. 1991; Hovden et al. 2016). PLD modulates the nuclei site and CDW to the energetically stable state (Hovden et al. 2016). Modified domains can be observed in high-resolution imaging and diffraction patterns. Hovden et al. (2016) investigated the visibility of commensurate structure projection images of 1 T-TaS_2_ PLD in 65 layers by using HAADF imaging. Without the ordering of PLD in the z-axis direction, the superlattice could not be seen in the projection image. This visible ordering in the projection image supported the c-axis ordering of the CDW suggested in the free-energy calculations (McMillan 1975).

**Fig. 3:**
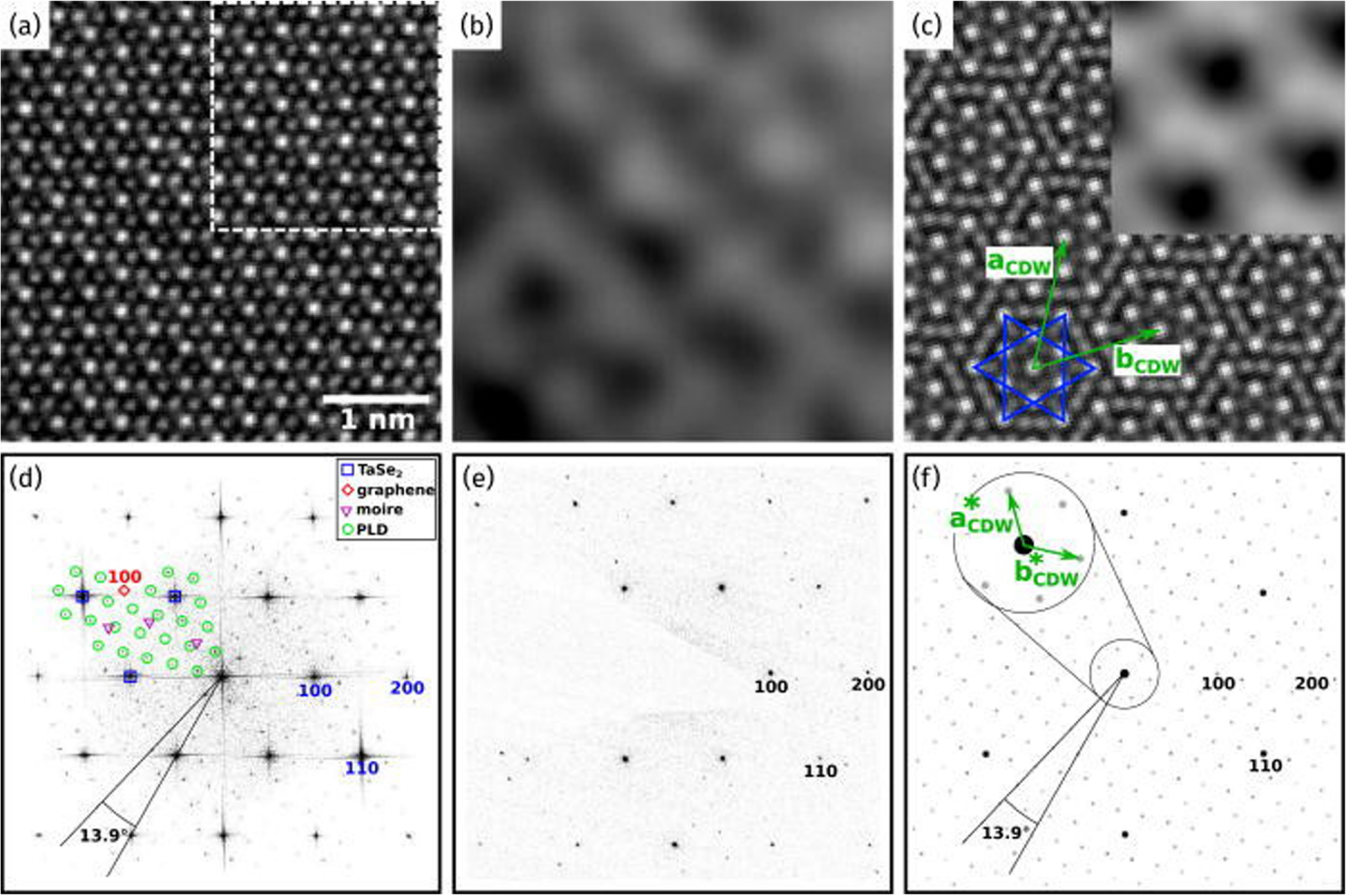
**a** Experimental AC-HRTEM 1 T-TaSe2/graphene heterostructure at 80 kV. **b** Contrast variation due to the CCDW/PLD. **c** Simulated AC-HRTEM image of the CCDW/PLD in a single 1 T-TaSe2 layer. The Star of David structure is marked in blue. **d** The Fourier transformation of image (**a**) showing the PLD structure peaks. **e** Visible satellite spots of the CCDW/PLD. **f** Simulated kinematic electron diffraction pattern of the CCDW/PLD in a single 1 T-TaSe2 layer. Reprinted from Börner et al. (2018) (*Appl. Phys. Lett.* 113, 173,103) with *Applied Physics Letters*’ permission

Moreover, the Fourier transform data of the PLD structure imaging indicates information that is not very clear in HRTEM imaging (Fig. 3) (Börner et al. 2018). The 1 T-TaSe_2_ monolayer/graphene heterostructure CDW state pattern was obscure in the HRTEM image as shown in Fig. 3. However, in the fast Fourier transform (FFT) image, the PLD peaks are easily discerned, and the 1 T-TaSe_2_ proved to be commensurate to the bulk state. This result confirmed the stability of CDW in the monolayer 1 T-TaSe_2_ layer (Börner et al. 2018).

#### -in situ-based structural observation

**Fig. 4:**
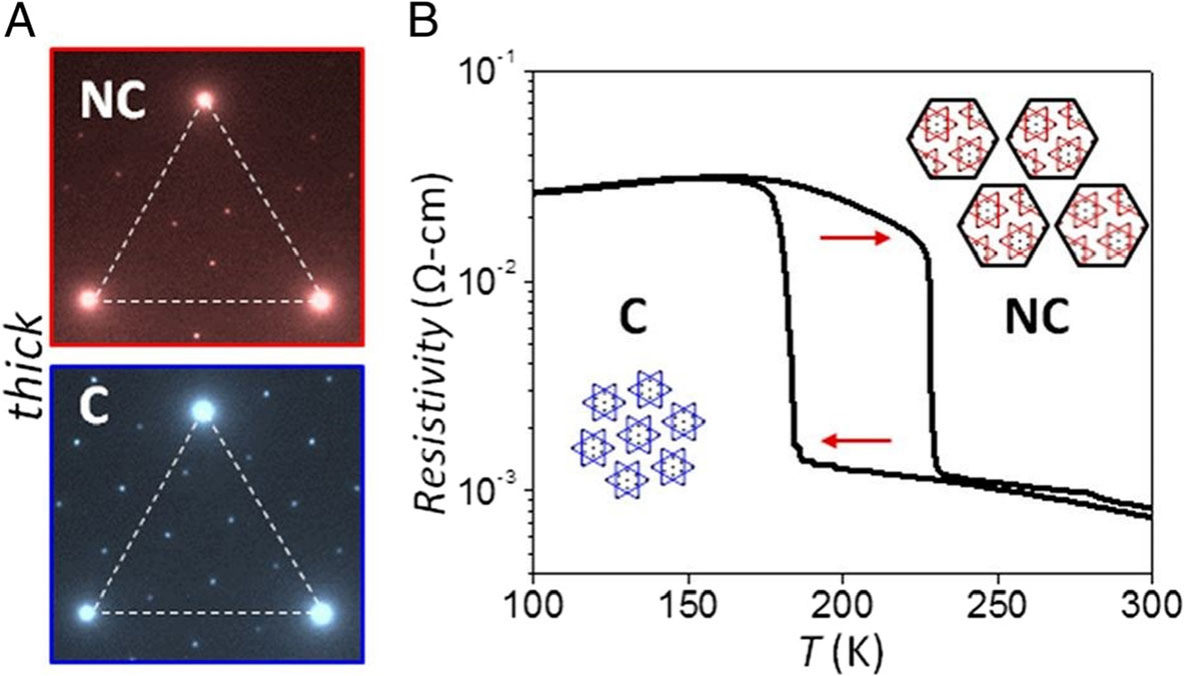
Nearly commensurate to commensurate phase transition in 1 T-TaS2 and CDW suppression by oxidation in thin flakes. **a** TEM diffraction images of 1 T-TaS2 at 295 K (red, NC phase) and 100 K (blue, **c** phase). Weaker peaks are due to CDW distortion. **b** Resistivity vs. temperature of bulk 1 T-TaS2 crystal around the first-order, NC-C transition. Reprinted from Tsen et al. (2015) (Proc. Natl. Acad. Sci. 112, 15,054–15,059) with National Academy of Sciences of Prospects of the United States of America’s permission

In situ TEM experiments probe structural changes under specific conditions such as heating, electrical biasing, and cooling. The PLD/CDW observations are an example of a cryo-in situ experiment. In the experiment, the temperature was decreased until the transformation of nearly commensurate into commensurate structures occurred (Fig. 4) (Hovden et al. 2016; Börner et al. 2018; Tsen et al. 2015). In situ heating experiments on 2D materials can illustrate the mechanisms of structural changes according to the temperature changes. Fei et al. (2016) envisaged the structural transformation mechanisms from precursor to vertical MoS_2_ layers by using a heating holder. Moreover, the structural orientation changes from vertical to horizontal were identified by HRTEM and SAED imaging.

### Observation of the microstructure of 2D materials in combination with electric measurements

The alteration in the twist angle between the layers in a van der Waals structure is applied for electronic characteristic tuning in 2D materials (Ju et al. 2017; Cao et al. 2018; Ke et al. 2019; Yoo et al. 2019). The bilayer twist method in graphene shows charge carrier density tunability with the precisely adjusted interlayer angle (Li et al. 2010; Kim et al. 2016; Cao et al. 2018). Recently, a 2D superlattice structure, called the magic angle (1.1°) twisted bilayer graphene (MA-TBG), showed a flat band near zero Fermi energy, and the insulating property was revealed (Cao et al. 2018). Insulating states resulted from the competition of Coulomb energy and quantum-like energy and were consistent with a Mott-like insulating state. This state can be changed into a superconducting state with electron doping and at temperatures reaching 1.7 K (Cao et al. 2018). Moreover, the TBG superlattice showed a quasi-periodic moiré pattern, and the moiré potential trapped the interlayer excitons, which functioned as the charge carriers (Woods et al. 2014; Seyler et al. 2019).

Yoo et al. (2019) showed the atomic and electronic reconstruction at the van der Waals interface in twisted bilayer graphene. Twisted graphene bilayers changed from an incommensurate domain to a strong commensurate domain with the gradual change in the angle (Yoo et al. 2019). Similar phenomena were reported in different 2D van der Waals bilayers and proved by scanning probe microscopy and scanning tunneling microscopy, and the results showed surface reconstruction at the interface (Woods et al. 2014). Yoo et al. (2019) obtained the TEM dark-field image and diffraction pattern and compared the experimental result with the simulations in both reconstructed and unreconstructed states. The satellite peaks shown in the experiments were not observed in the unconstructed surface, which proves the atomic reconstruction in twisted bilayer graphene. In the commensurate state at the magic angle of 1.1°, the calculation of the unreconstructed state interlayer band structure of the twisted bilayer graphene did not comply with the real data. The experimental data showed better consistency with the reconstruction band structure. Moreover, the traverse displacement field applied in the moiré patterns proved the 1D channel formation due to atomic reconstruction (Yoo et al. 2019). These experimental results demonstrated surface reconstruction between the bilayers.

In PLD, the commensurate and incommensurate structures are also related to the electrical resistivity; therefore, microstructural analysis combined with the electric measurements was conducted. For the functional use of 1 T-TaS_2_, a persisting and nearly commensurate structure throughout the temperature changes is necessary for better electric device conductivity. Tsen et al. (2015) measured the resistivity of 1 T-TaS_2_ of different thicknesses with changes in temperature. The resistivity-temperature curve of the thin sample showed the persistence of the nearly commensurate state. TEM diffraction peaks from 2 nm and 12 nm layers were compared and demonstrated the disappearance of commensurate peaks in the 2 nm sample. This result was consistent with the electronic measurements (Tsen et al. 2015). These experiments proved the importance of TEM imaging and diffraction in correlation with electrical property measurements to better understand the origin of material properties on an atomic scale.

## Conclusions

Because the electronic and optical properties of 2D materials can be tuned with structural changes, previous investigations focused on microstructural analyses. TEM studies on 2D materials demonstrated the defects, interstitial atomic formations, and other structural scale distortions and atomic configurations. Beginning with low-voltage electron beams in which the 2D material sample remains stable, the progress of research has revealed many mechanisms in the formation of structural changes. Recent microstructural studies have assessed the structural distortion-induced electrical properties. These studies combined TEM analyses with other electrical measurements, e.g., conductivity measured using applied traverse field voltage and resistivity measured by voltage changes. These results have been supported by prior studies on 2D material electronic coupling and other unique electronic characteristics induced by microstructural changes.

Although progress has been made in this area, 2D material research continues to advance. Combined with technical developments in TEM, which have led to sub-Angstrom resolution analysis in low-voltage TEM, 2D material research will become a better understood field with the potential for exciting discoveries.

## Data Availability

Not applicable.

## Abbreviations

2D materials: Two-dimensional materials
CDW: Charge density wave
FFT: Fast Fourier transform
HAADF: High-angle annular dark field
HRTEM: High-resolution transmission electron microscopy
MA: Magic angle (1.1°)
PLD: Periodic lattice distortion
SAED: Selected area electron diffraction
STEM: Scanning transmission electron microscopy
TBG: Twisted bilayer graphene
TEM: Transmission electron microscopy
TMD: Transition metal dichalcogenides
